# Topographical and Biological Evidence Revealed FTY720-Mediated Anergy-Polarization of Mouse Bone Marrow-Derived Dendritic Cells *In Vitro*

**DOI:** 10.1371/journal.pone.0034830

**Published:** 2012-05-31

**Authors:** Xiangfeng Zeng, Tong Wang, Cairong Zhu, Xiaobo Xing, Yanxia Ye, Xinqiang Lai, Bing Song, Yaoying Zeng

**Affiliations:** 1 Institute for Tissue Transplantation and Immunology, Jinan University, Guangzhou, China; 2 Institute of Life and Health Engineering, Jinan University, Guangzhou, China; 3 Department of Chemistry, Jinan University, Guangzhou, China; 4 Guangzhou Women and Children’s Medical Center, Guangzhou, Guangdong, China; 5 MOE Key Laboratory of Laser Life Science and Institute of Laser Life Science, South China Normal University, Guangzhou, China; Hannover Medical School, Germany

## Abstract

Abnormal inflammations are central therapeutic targets in numerous infectious and autoimmune diseases. Dendritic cells (DCs) are involved in these inflammations, serving as both antigen presenters and proinflammatory cytokine providers. As an immuno-suppressor applied to the therapies of multiple sclerosis and allograft transplantation, fingolimod (FTY720) was shown to affect DC migration and its crosstalk with T cells. We posit FTY720 can induce an anergy-polarized phenotype switch on DCs *in vitro*, especially upon endotoxic activation. A lipopolysaccharide (LPS)-induced mouse bone marrow-derived dendritic cell (BMDC) activation model was employed to test FTY720-induced phenotypic changes on immature and mature DCs. Specifically, methods for morphology, nanostructure, cytokine production, phagocytosis, endocytosis and specific antigen presentation studies were used. FTY720 induced significant alterations of surface markers, as well as decline of shape indices, cell volume, surface roughness in LPS-activated mature BMDCs. These phenotypic, morphological and topographical changes were accompanied by FTY720-mediated down-regulation of proinflammatory cytokines, including IL-6, TNF-α, IL-12 and MCP-1. Together with suppressed nitric oxide (NO) production and CCR7 transcription in FTY720-treated BMDCs with or without LPS activation, an inhibitory mechanism of NO and cytokine reciprocal activation was suggested. This implication was supported by the impaired phagocytotic, endocytotic and specific antigen presentation abilities observed in the FTY720-treated BMDCs. In conclusion, we demonstrated FTY720 can induce anergy-polarization in both immature and LPS-activated mature BMDCs. A possible mechanism is FTY720-mediated reciprocal suppression on the intrinsic activation pathway and cytokine production with endpoint exhibitions on phagocytosis, endocytosis, antigen presentation as well as cellular morphology and topography.

## Introduction

Dendritic cells (DCs) represent a typical cell type participating in the “dual-edge sword” in immunity. In addition to serving as professional antigen-presenting cells, involved in beneficial immuno-protection, they are also key effector cells in a variety of detrimental chronic and acute inflammations. For example, chronic lesion formation in atherosclerosis was found to be etiologically relevant to DC-induced proinflammatory cytokine production, especially in terms of synergistically inflammatory attacks on the vascular walls caused by hypercholesterolemia in blood and microbial antigens on endothelial cells [1]–[4]. This is comparable with more specific findings in acute bacterial infections [5], [6]. When exposed to gram-negative bacteria, the endotoxin-induced primary activation of DCs is frequently followed by the formation of desensitized DCs, known as LPS-tolerance DCs. This mechanism was found pivotal in sepsis survivors as a negative-feedback mechanism to prevent acute endotoxic shock [5], [6].

These DC-relevant chronic and acute inflammations play important roles in a number of autoimmune and allergy diseases as aberrant antigen presentation and/or DC response involves [7]. It was shown self-protein-loaded DCs can trigger autoimmunity via TLR3 and TLR9 ligations in the development of autoimmune myocarditis [8]. Similarly, in multiple sclerosis (MS), self-antigen responsive T cells can be sufficiently primed by DCs to initiate central nervous system (CNS) inflammation, responsible for disease progression [9]. This mechanism is further confirmed by the observation showing the prolonged life span of DCs is associated with chronic lymphocyte activation and systemic autoimmune manifestations [10], [11]. Comparably, these DC-relevant inflammations are observed in various allergies. Particularly, abnormal response of DCs to thymic stromal lymphopoietin is deemed a key mechanism in priming the T helper 2 (Th2) cells to produce inflammatory cytokines in allergic diseases, such as asthma, allergic rhinitis and food allergies [12]. Taken these aspects together, DC-induced inflammation is one of the common nodes for therapy of the above-mentioned human diseases; and as proposed by numerous investigations, proper modulation of DCs to suppress such inflammations is a useful therapeutic strategy (reviewed in [7], [13]).

As a newly approved drug on MS treatment, fingolimod (FTY720) was firstly synthesized by chemically modifying myriocin, a metabolite of *Isaria sinclairii*, used as a component in traditional Chinese medicine [14]. The major therapeutic mechanism of FTY720 is to sequester autoimmune T cells in the secondary lymphoid tissues and reduce the T cell infiltration in CNS [15]–[18].

Indeed, the initial efforts on the development of FTY720 were largely confined in the immune-suppressor effects that are demanded in tissue and organ transplantations. Although its efficacy was confirmative, the high dose of application was a concern, leading to an investigative diversion to treatments of other diseases, such as MS and cancer (reviewed in [18]). However, plentiful knowledge was acquired from early studies, especially in terms of possible functions of FTY720 on DCs. In a subtle study, with a mixed lymphocyte reaction (MLR) model, FTY720-treated human DCs exhibited reduced antigen presentation function and altered cytokine production after stimulation with CD40 ligand-expressing cells [19]. In addition, FTY720 was found to block DC trafficking that is relevant to its immunosuppressive effects [20].

With the rationale above, we posit the effects of FTY720 on DCs include an anergy-driven polarization mechanism, especially upon endotoxic activation. Addressing this question is clinically relevant to propose an expansion of FTY720’s application to modulate more generalized chronic and acute inflammations, targeting at DCs. Therefore, we employed a lipopolysaccharide (LPS)-induced mouse bone marrow-derived dendritic cell (BMDC) activation model and demonstrated direct effects of FTY720 to induce anergy-polarization of BMDCs, for the first time, in terms of nanostructure, biological functions and possible mechanisms.

## Results

### FTY720 Changes the Surface Phenotypes of BMDCs Upon LPS Activation

It has been shown FTY720 does not significantly change phenotypes of both human immature and mature DCs isolated from peripheral blood, however only decreases CD18 expression [19]. We posit FTY720 alone should have minimal effects on the maturation of BMDC; however upon LPS treatment, FTY720 is able to suppress the LPS-induced maturation. All experiments mentioned in this section were repeated for 3 times, independently.

**Figure 1:**
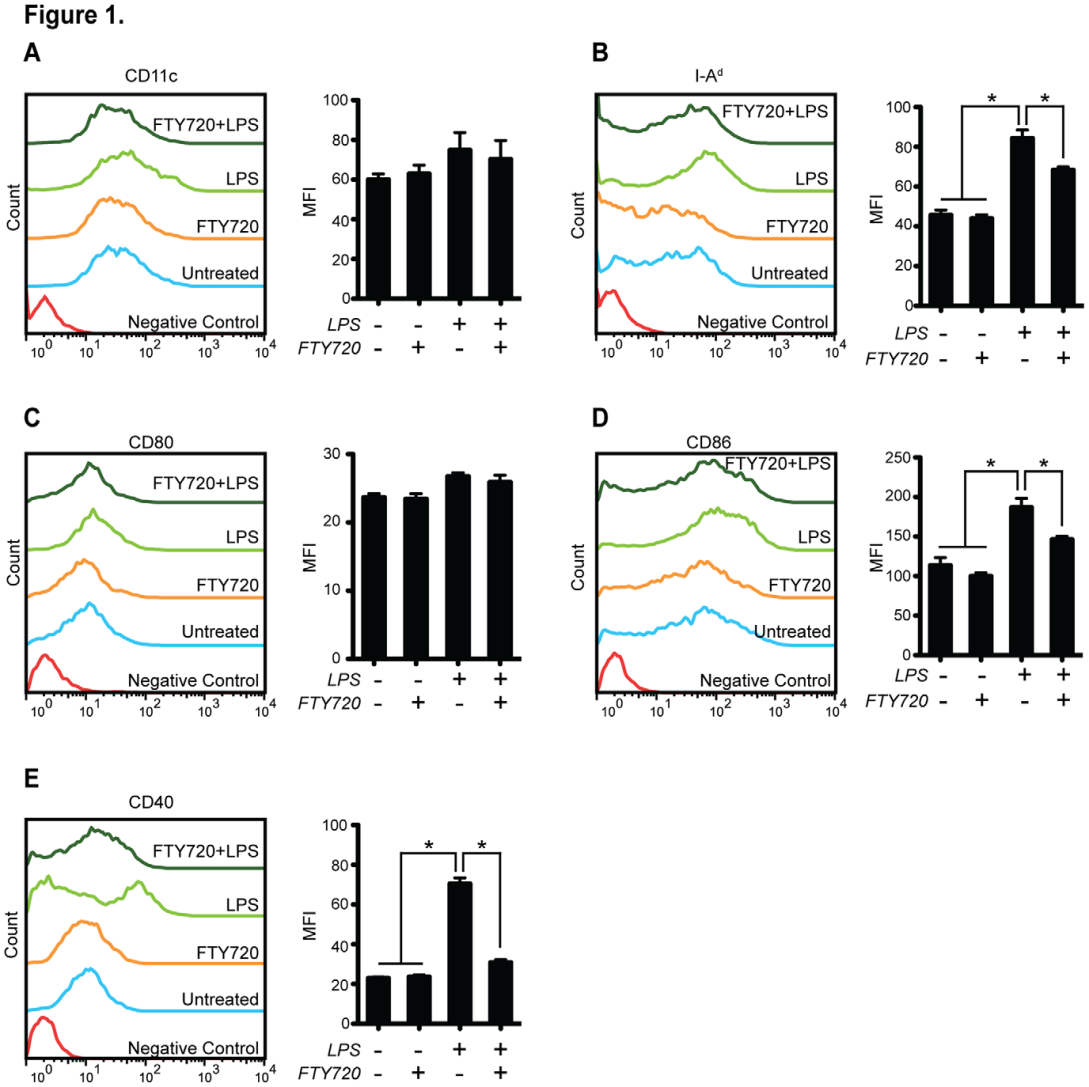
FTY720 alters the surface phenotype of mouse bone marrow-derived dendritic cells upon LPS activation. Expression of CD11c (**A**), MHC II molecule I-A^d^ (**B**), CD80 (**C**), CD86 (**D**) and CD40 (**E**) was shown in their respective panels. For these assays, mouse bone marrow cells were differentiated for 6 d to prepare BMDCs that were exposed to LPS (1 µg/mL) and/or FTY720 (500 nM) for additional 24 h. Representative results out of three independent experiments were shown. The mean fluorescence intensities (MFI) of each marker were analyzed for statistical difference. *P<0.05, n = 3.

After being co-treated by granulocyte macrophage colony-stimulating factor (GM-CSF) and IL-4, mouse bone marrow cells were differentiated into BMDCs, showing (95.9±1.7)% positive for the macrophage/DC lineage marker CD11c (Figure 1A). The expression of CD11c surface molecules was not significantly changed in either the LPS- and/or the FTY720- treated groups (Figure 1A).

LPS treatment was observed to induce typical maturation phenotypes of BMDCs (Figure 1B–E). First, LPS-treated BMDCs showed significant up-regulation of MHC II molecule, I-A^d^ (Figure 1B), and the expression of co-stimulatory molecules, including CD86 (Figure 1D) and adhesion molecule CD40 (Figure 1E) (*P*<0.05, n = 3). Thus, the positive control (LPS-activated mature DC) and negative control (untreated immature DC) for this experiment were validated by these data. No significant difference was observed for CD80 expression between different groups (Figure 1C). Compared with the untreated group, FTY720 exhibited no considerable changes on the surface molecules at the major experimental concentration of 500 nM (Figure 1 A–E). However, comparing with the LPS-treated groups, FTY720 suppressed LPS-induced BMDC maturation by significantly alleviating the expression of I-A^d^ (Figure 1B), CD86 (Figure 1D) and CD40 (Figure 1E) (*P*<0.05, n = 3).

### FTY720 Reverses the Morphological and Topographical Changes in LPS-activated Mature BMDCs

**Figure 2:**
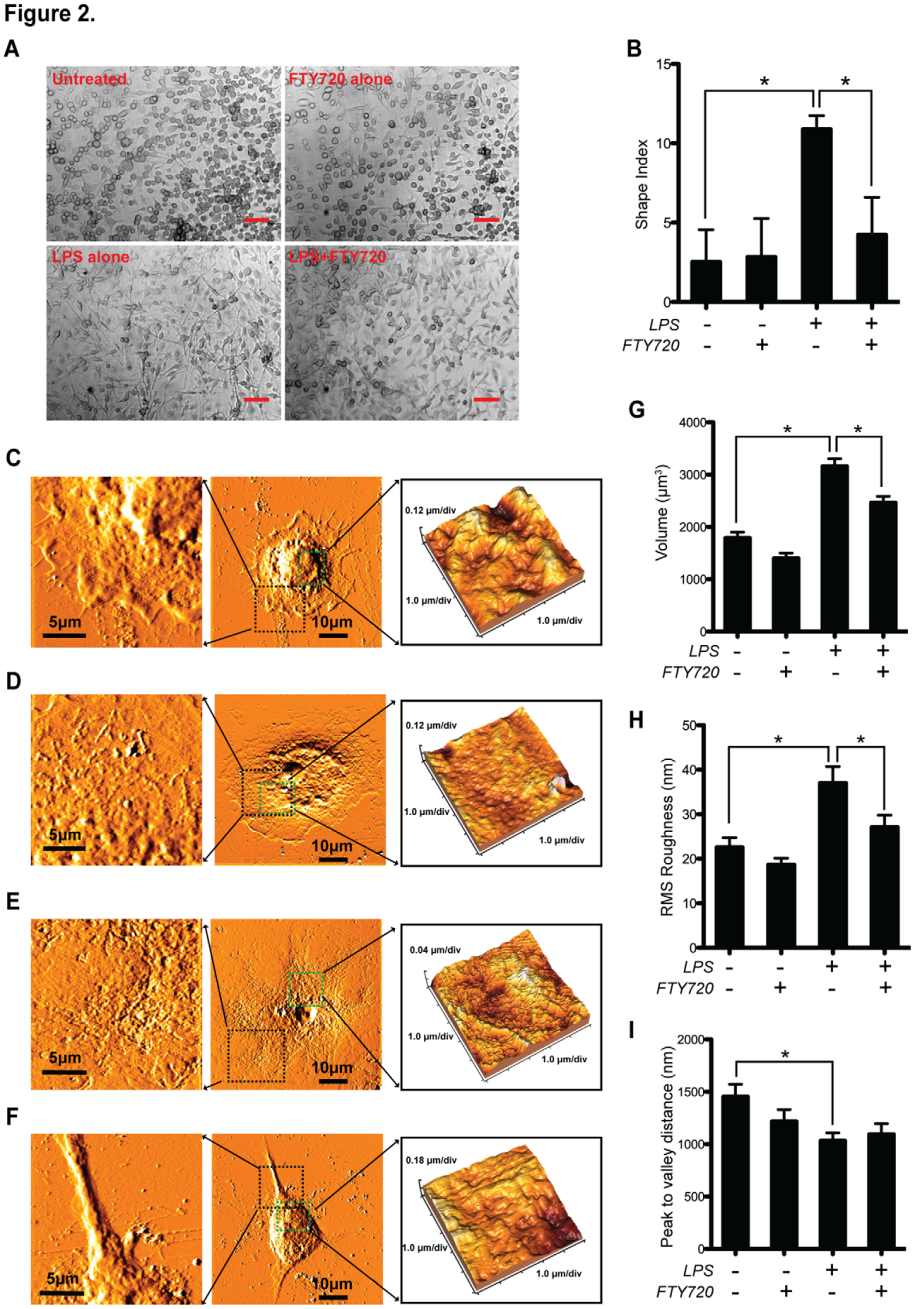
Topographical and morphological changes of BMDCs upon LPS-activation are reversible by FTY720. (**A**) Morphologies of LPS- and/or FTY720- treated BMDCs. Day 6 BMDCs were exposed to LPS (1 µg/ml) and/or FTY720 (500 nM) for additional 48 h, followed by microscopic observations. Scale bar  = 20 µm. (**B**) Statistical results on the shape indices of each group. *P<0.05, n = 20 (20 cells randomly selected from high power fields from 3 separate experiments). Atomic force microscope observations on the untreated (**C**), FTY720 alone (**D**), LPS alone (**E**) and LPS+FTY720- (**F**) treated groups were shown as representative images. Enlarged areas were indicated in squares in dark and green, respectively. The statistical results of the cell volume (**G**), RMS roughness (**H**) and peak to valley distance (**I**) are analyzed and shown in histograms. Data are shown as mean ± SEM. *P<0.05, n = 10 (10 cells randomly selected from 3 separate experiments).

As shown in Figure 1, FTY720 changed maturation-relevant surface markers of BMDC upon LPS activation. We further examined these suppressive functions of FTY720 at a nanostructure level on BMDCs during the inflammatory response upon LPS-activation BMDCs (Figure 2).

Treated by LPS alone, BMDCs showed increased elongation morphologies, compared with the untreated and the FTY720 alone group; however these LPS-induced elongations were suppressed by FTY720 (Figure 2A). We next calculated the cell shape index (major axis/minor axis) of each group [21]. LPS treatment increased the shape index by approximate 4 folds, compared with both the untreated and the FTY720 alone group, respectively (*P*<0.05, n = 20; Figure 2B). In the LPS+FTY720 group, the shape index was significantly reduced to the level close to the untreated group (*P*<0.05, n = 20; Figure 2B).

We previously employed atomic force microscopy (AFM) as an efficient way to topographically investigate the nanostructural changes in different cell types, especially in DCs [22]–[24]. A similar evaluation strategy was used in this study (Figure 2C–F). Consistent with the shape index results, cells in the untreated and the FTY720 alone-treated group shared a round-shaped morphology covering approximately 40×40 µm^2^ growth area (Figure 2C and 2D), however the elongated cells are predominant in the LPS-treated group with a stretching morphology covering around 70×70 µm^2^ growth area (Figure 2E). In the LPS+FTY720 treated group, these LPS-mediated DC morphological changes were reversed to a similar level to the untreated BMDCs (Figure 2F).

Topographically, FTY720 alone cannot significantly down-regulate cell volume (Figure 2G), root square mean (RSM) roughness (Figure 2H) and the peak to valley distance (Figure 2I) of BMDCs. In contrast, compared with the untreated group, these parameters were increased by approximately 1.5 folds (*P*<0.05, n = 10) (Figure 2G–I) in the LPS-treated group, accompanied by a significant reduction of cellular peak to valley distance (Figure 2I) (*P*<0.05, n = 10). Both cell volume and RMS roughness upon LPS activation were suppressed significantly by co-treatment with FTY720 (*P*<0.05, n = 10; Figure 2G and 2H).

### Scanning Electron Microscopic Validation

**Figure 3:**
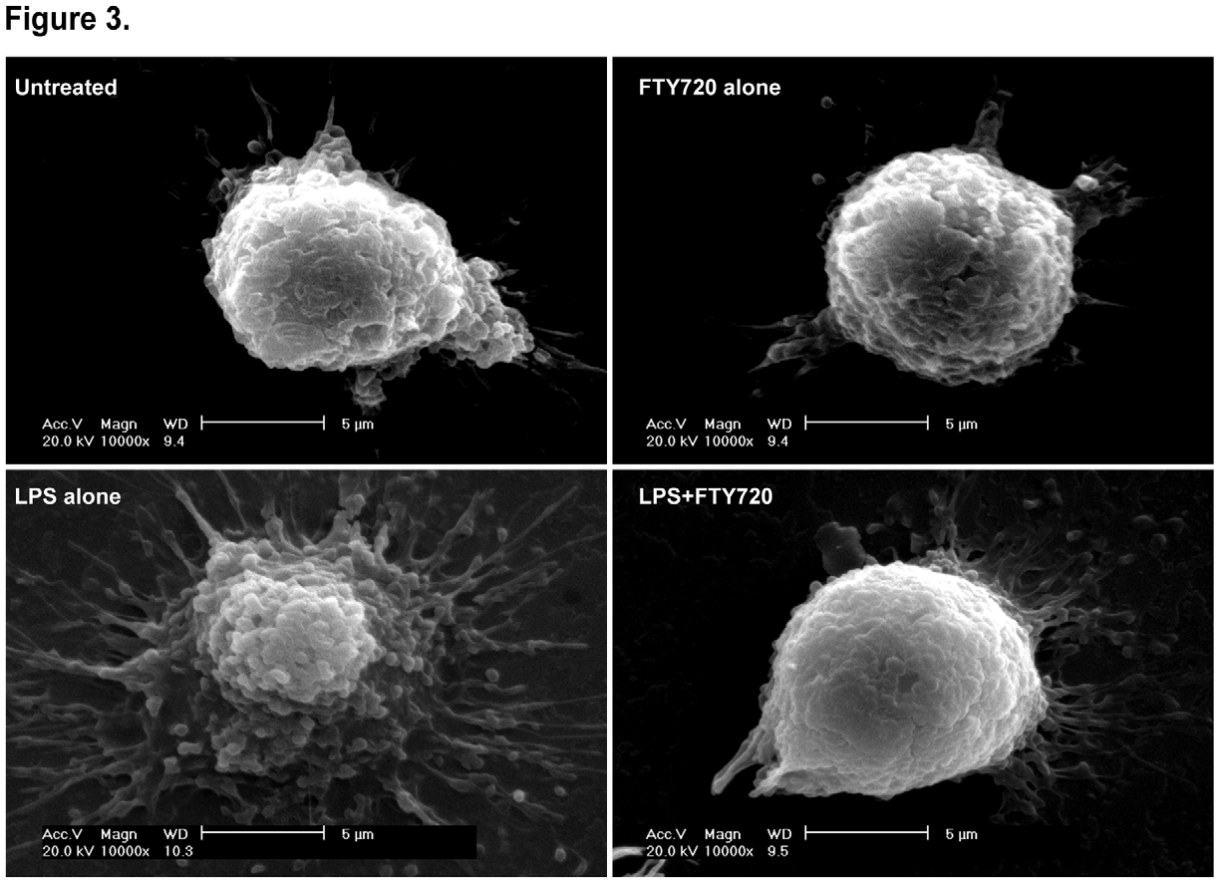
Scanning electron microscopic observation. Day 6 BMDCs were treated with LPS (1 µg/mL) and/or FTY720 (500 nM) for additional 48 h before SEM observation. Scale bar = 5 µm.

To validate the cell surface roughness changes observed in AFM experiments, we further performed scanning electron microscopy (SEM) (Figure 3). Consistently, dendrites and largely round-shaped BMDCs were observed in the untreated and FTY720 alone-treated groups. LPS-activated mature BMDCs exhibited remarkably increased number of dendrites, vesicle-abundant surface structures and dendrite morphologies. However, in the LPS+FTY720-treated group, most cells tended to be polarized to immature BMDC-like morphologies. This is another visualization confirmation on the topographical results obtained from AFM experiments.

### Cell Viability Assay

**Figure 4:**
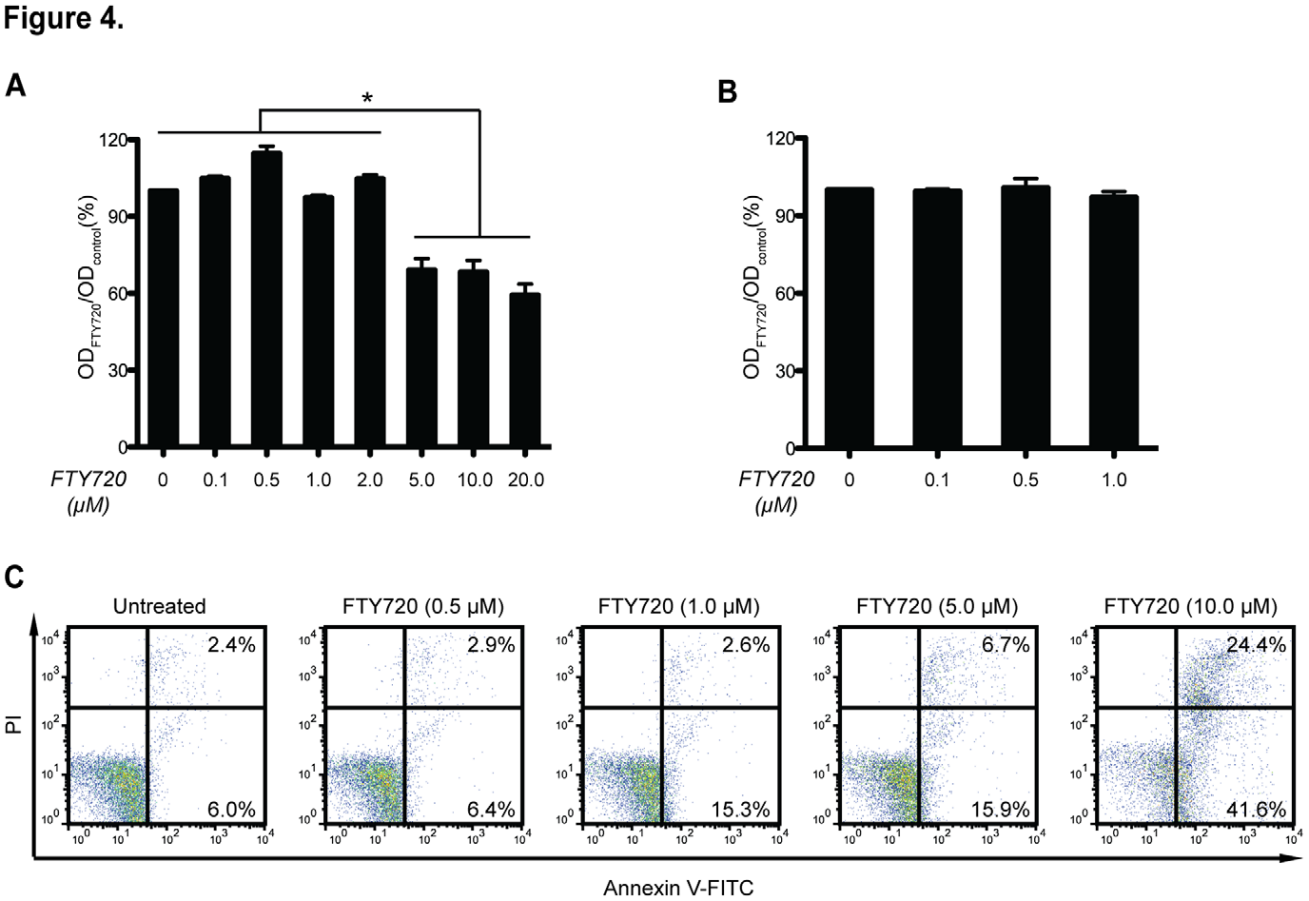
Cell viability determination. (**A**) MTT assays on the bone marrow cells. Mouse bone marrow cells were isolated and exposed to FTY720 with different concentrations for 48 h. Cytotoxicity was indicated by the ratio of OD_FTY720_ to OD_control_. Data are shown as mean ± SEM. *P<0.05, n = 3. (**B**) MTT assays on BMDCs. Day 6 BMDCs were treated with FTY720 at concentrations of 0, 0.1, 0.5 and 1.0 µM for 48 h. Data are shown as mean ± SEM. *P<0.05, n = 3. (**C**) Apoptosis assays on BMDCs. The cell death rates of BMDCs was determined by Annexin V-FITC and PI staining flow cytometry. Cells were treated with FTY720 for 48 h at concentrations ranged from 0.5 to 10 µM.

As we observed significantly suppressive modulation of FTY720 on BMDCs, especially in terms of LPS mediated activation, we have to rule out the possible cytotoxicity effects of the drug before providing further biological mechanisms. We addressed this question by cell viability assays on FTY720-treated bone marrow cells and BMDCs, respectively (Figure 4). No cytotoxicity was observed in mouse bone marrow cells treated by FTY720 at concentrations of 0, 0.1, 0.5, 1.0 and 2.0 µM, however higher concentrations of FTY720 could induce significant cytotoxicity (*P*<0.05, n = 3; Figure 4A). Regarding BMDCs, FTY720 did not show significant cytotoxicity effects at all tested concentrations in the MTT assay (Figure 4B). With concentrations at 1.0, 5.0 and 10 µM, the apoptosis/necrosis rates of BMDCs were observed greater than 17%, 23% and 65%, respectively (Figure 4C). However, FTY720 did not induce BMDC cell death at the concentration of 500 nM, which is the primary concentration used in most of the experiments in this study (Figure 4C).

### FTY720 Suppresses the Cytokine Production in BMDCs Upon LPS Activation

**Figure 5:**
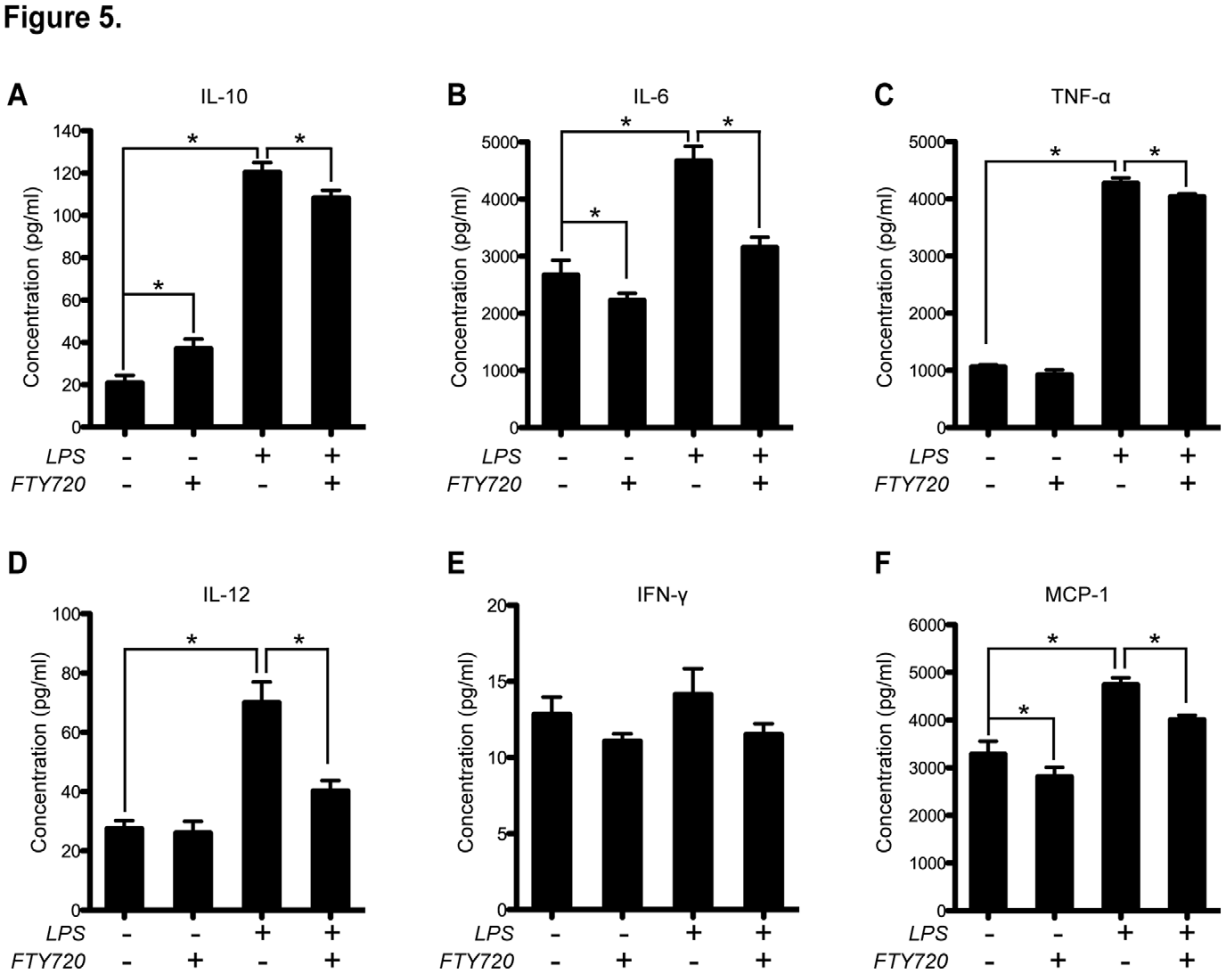
Cytometric Bead Array analysis. CBA assays on IL-10 (**A**), IL-6 (**B**), TNF-α (**C**), IL-12 (**D**), IFN-γ (**E**) and MCP-1 (**F**) were shown in their respective panels. For these assays, Day 6 BMDCs were treated with LPS (1 µg/mL) and/or FTY720 (500 nM) for 24 h. The culture supernatants of each group were subjected to CBA analysis by flow cytometry. Data are shown as mean ± SEM. *P<0.05, n = 3. The cytokine concentrations were calculated from the standard curve (*R* ^2^>0.95) via data analysis by a four parameter linear fitting program provided by the manufacturer.

From the topographical and morphological experiments, we observed abundant secretory vesicle-like structures in LPS-treated BMDCs, which were considerably different from other experimental groups. We posit this could imply a secretory suppression mechanism in FTY720-modulated BMDCs. Thus, we tested the cytokine concentrations by cytometric bead array (CBA) analysis in the culture supernatants to address this question (Figure 5). Three independent experiments were performed on these experiments.

First, FTY720 alone showed an anti-inflammation phenotype switch, compared with the untreated BMDCs, evidenced by significant up-regulation of IL-10 secretion (*P*<0.05, n = 3; Figure 5A), as well as significant down-regulation of IL-6 and MCP-1 production (*P*<0.05, n = 3; Figure 5B and 5F, respectively). Second, BMDCs showed typical activation phenotypes, exhibiting approximately 1.5–6 fold increases of cytokine production upon LPS activation, including IL-10, IL-6, TNF-α, IL-12 and MCP-1 (*P*<0.05, n = 3; Figure 5A–D and 5F, respectively). Third, co-treatment with FTY720 suppressed all these LPS-induced cytokine productions, significantly (*P*<0.05, n = 3; Figure 5A–D and 5F). Finally, no significant difference was observed for IFN-γ production in all experimental groups (Figure 5E).

### FTY720 Suppresses Phagocytosis and Endocytosis of BMDCs Accompanied by Inhibition of CCR7 and NO Production

**Figure 6:**
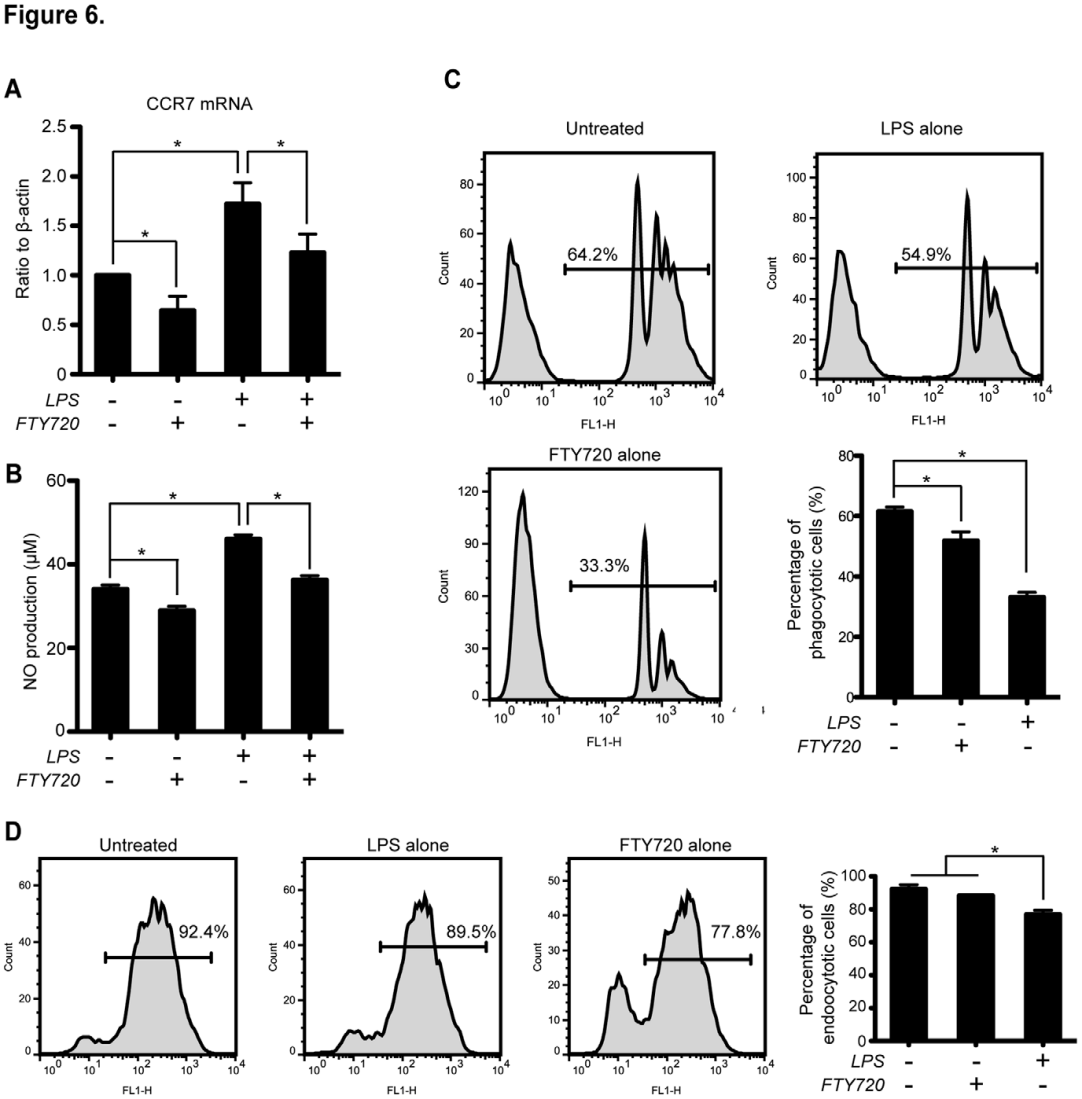
CCR7 transcription, NO production as well as phagocytosis and endocytosis of BMDCs. (**A**) CCR7 mRNA production analysis. Day 6 BMDCs were treated with LPS (1 µg/mL) and/or FTY720 (500 nM) for 24 h. Total mRNA were extracted, reverse transcripted and quantified by the real-time PCR. *P<0.05, n = 3. (**B**) Nitric oxide production assays. Supernatants of cells with similar treatments were obtained and analyzed by diazotization reaction and colorimetry. *P<0.05, n = 3. (**C**) Phagocytosis assay of BMDCs. Day 6 BMDCs were treated with LPS (1 µg/ml) alone or FTY720 (500 nM) alone for 24 h and subjected to fluorescence-conjugated beads uptake assays. Representative of results on the percentage of phagocytes were shown in each flow chart. Statistical analysis on the percentage of phagocytotic cells were presented as mean ± SEM. *P<0.05, n = 3. (**D**) Endocytosis assay of BMDCs. Similar experimental design to (**C**) were employed. Cells were treated with FITC-conjugated dextran and analyzed by FACS. Representative results on the percentage of endocytotic cells were shown. Statistical data were presented as mean ± SEM. *P<0.05, n = 3.

It is known that LPS up-regulates CCR7 expression [25], which is a versatile molecule relevant to migration, maturation and endocytosis of DC [26]–[28]. This activation is in part due to nitric oxide (NO) induction, although ambiguous reports indicated diverted functions of NO when different doses or treatment durations were applied [29]. We posit FTY720 inhibit the LPS-mediated proinflammatory response partially by down-regulating phagocytosis, endocytosis, CCR7 and NO production. Therefore, these phenotypes were examined and all these experiments were performed for three times, independently (Figure 6).

Treatment with FTY720 alone was observed to be sufficient to down-regulate both CCR7 mRNA transcription and NO secretion of BMDCs, significantly (*P*<0.05, n = 3; Figure 6A and 6B). LPS stimulation significantly up-regulated CCR7 mRNA and NO production for approximately 1.5 folds as anticipated (*P*<0.05, n = 3; Figure 6A and 6B). However, both LPS-induced increases of CCR7 mRNA and NO production in BMDCs were significantly suppressed by co-treatment with FTY720 (*P*<0.05, n = 3; Figure 6A and 6B).

After 1 h exposure to fluorescent beads for phagocytosis assay, the immature BMDCs generally showed higher proportion of phagocytotic cells than the LPS alone and the FTY720 alone- treated cells (Figure 6C). Statistically, the total rate of the phagocytotic cells in the untreated group was (61.5±2.1)%, significantly higher than those in the LPS-treated (51.9±4.1)% and the FTY720 alone- treated (33.2±2.2)% groups (*P*<0.05, n = 3; Figure 6C).

We next analyzed fluid-phase endocytosis of BMDCs by tracing internalization of FITC-conjugated-dextran, a component of endosome [30]. The total rates of the endocytotic cells in the untreated and the LPS-treated groups were (92.3±2.7)% and (88.3±1.6)%, respectively, both significantly higher than the FTY720 alone-treated (76.3±2.6)% group (*P*<0.05, n = 3; Figure 6D).

### FTY720 Mitigates Specific Antigen Presentation Ability of BMDCs

**Figure 7:**
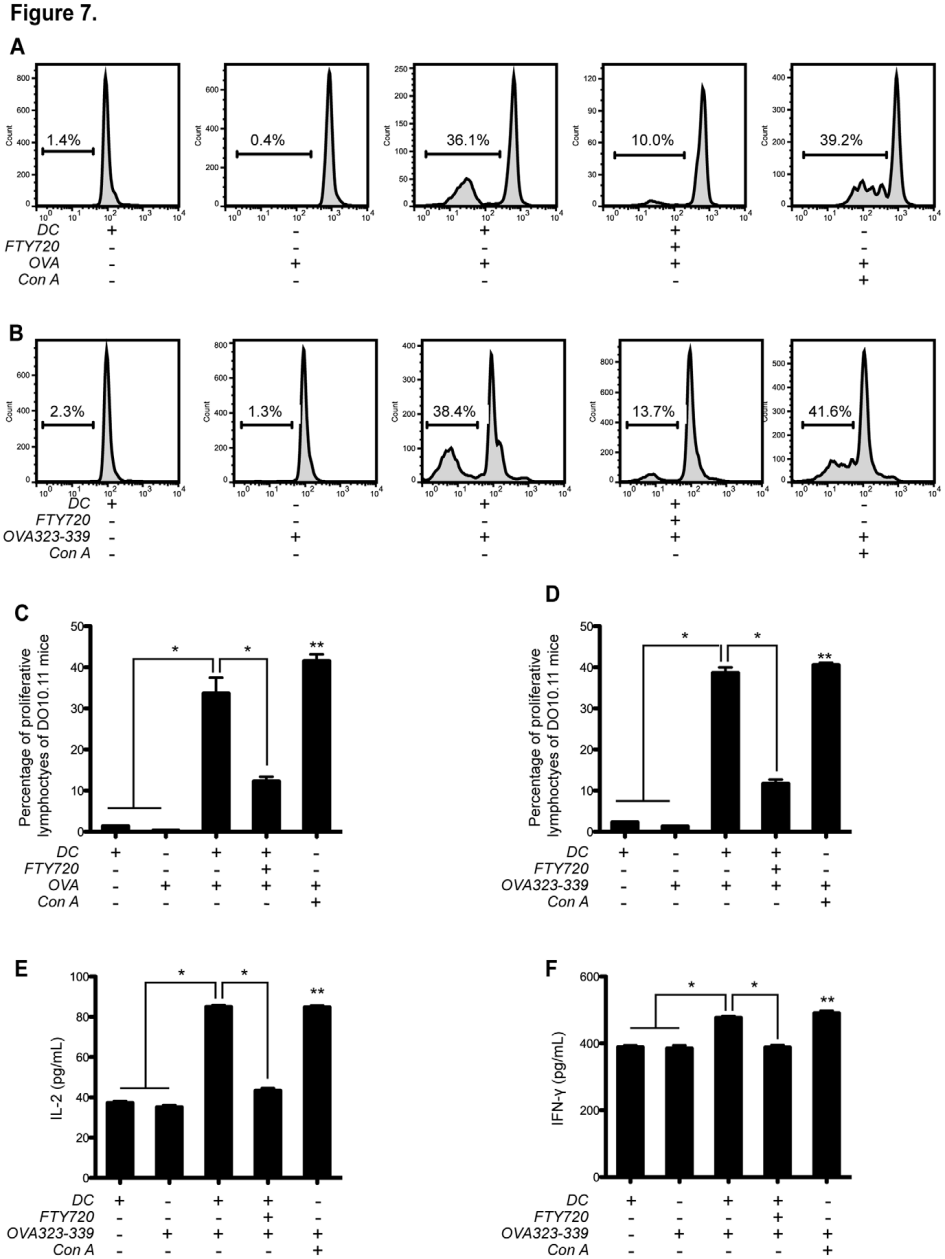
FTY720 suppresses the specific antigen presentation ability of BMDCs. (**A**) FTY720 impaired the ovalbumin (OVA) protein presentation ability of BMDCs. Lymphocytes from transgenic mice DO11.10 strain, producing T cells specifically recognizing OVA epitope at amino acid sequence from 323 to 339, were obtained from lymph node. Day 6 BMDCs pre-treated with or without FTY720 (500 nM) were employed to present OVA protein and co-cultivated with DO11.10 lymphocytes. T cell proliferation was subsequently analyzed by CFSE-staining flow cytometry; the fluorescence was detected at FL1 channel. A Con A-induced T cell proliferation model was adopted as a positive control. As negative controls, DO11.10 lymphocytes were exposed either to BMDCs with no presence of OVA, or to OVA with no BMDC co-cultivation. (**B**) Confirmative assays with OVA peptide 323–339. Similar experimental design to (**A**) was employed, however OVA peptide 323–339 was used. Statistical results of the percentages of proliferative OVA (**C**) and OVA peptide 323–339 (**D**) -responsive T cells were shown. (**E**) The production of supernatant IL-2. (**F**) IFN-γ secretion. Statistical data were presented as mean ± SEM. *P<0.05, n = 3. **P<0.05, n = 3, compared with the negative control groups.

As demonstrated above, maturation associated phenotypes, including topography, morphology, phagocytosis and endocytosis of BMDCs were down-regulated by FTY720. These phenomena imply a collective endpoint behavior of BMDC anergy to inflammation and adaptive immunity. Therefore, we hypothesize these anergy-polarized phenotypes should be accompanied by mitigated abilities of specific antigen presentation ability of BMDCs. Thus, we isolated lymphocytes from transgenic mice, DO11.10 strain, with transgenic T cell receptors that are reactive to ovalbumin (OVA) peptide 323–339 antigen, specifically. By testing the cell proliferation behavior of these OVA-specific T cells with CFSE-staining flow cytometry, the OVA presentation abilities of BMDCs with or without FTY720 treatment could be determined (Figure 7). Results were acquired from three independent experiments.

The DO11.10 mouse lymphocytes did not proliferate upon co-cultivation with BMDC with no presence of OVA treatment (Figure 7A&7B). Similarly, no lymphocyte proliferation was observed in the OVA- (Figure 7A) or OVA peptide 323–339- (Figure 7B) treated DO11.10 mouse lymphocytes groups. Both of these groups were served as negative control groups in the experiments of this section. As a positive control, DO11.10 lymphocytes can proliferate responsive to the stimulation concanavalin A (Con A) alone (Figure 7A&7B). Untreated BMDCs exhibited both OVA protein (Figure 7C) and OVA peptide 323–339- (Figure 7D) presentation ability by significantly promoting proliferation of DO11.10 mouse lymphocytes (*P*<0.05, n = 3). If BMDCs were pre-treated with FTY720, this antigen presentation ability was significantly reduced (*P*<0.05, n = 3; Figure 7C&7D).

To determine the feature of T cell polarization in OVA peptide 323–339-induced DO11.10 lymphocyte proliferation, we tested the production of two Th1 cytokines, IL-2 (Figure 7E) and IFN-γ (Figure 7F). Upon OVA peptide 323–339 treatment, the production of both cytokines were significantly up-regulated in the untreated BMDC co-cultivation group, compared with the two negative control groups (*P*<0.05, n = 3; Figure 7E&7F). However, if co-cultivated with FTY720-treated BMDCs, this up-regulation of the two cytokines was significantly alleviated (*P*<0.05, n = 3; Figure 7E&7F).

## Discussion

The overall results of this study suggested FTY720 induces an anergy-polarized effect on BMDCs, exhibiting molecular, topographical, morphological and functional changes. As a widely used Chinese herbal medicine, *I. sinclairii* is believed to be able to put down “the fire”, an ancient way in China to describe inflammations in human body [31]. Actually, this was found largely due to the immune-suppressive functions of myriocin, the pre-drug of FTY720, abstracted from *I. sinclairii* [32]. Conceptually, our findings in this study imply a consistent application of FTY720 to chronic and acute inflammatory diseases, however revealed DCs are a direct target cell type for calming down these aberrant inflammations. Therefore, we provided an insight of FTY720-induced DC modulation, not confined to the field of transplantation studies.

We demonstrated FTY720 alone does not affect the surface maturation markers of BMDCs, including CD11c, MHC II molecules, CD80, CD86 and CD40. Indeed, these results are comparable with a number of investigations on FTY720 and DCs [19], [33], [34], thus also serving as a confirmation of valid preparation of BMDCs for the entire experiments in this study. These unimpaired surface markers are also indicators of non-toxic effects of FTY720 on BMDCs, which was confirmed by the subsequent cell viability assays, especially validating that the 500 nM experimental concentration of FTY720 did not induce cytotoxicity on BMDCs. These phenotypic profiling and non-cytotoxic results served as a valid basis of following functional investigations.

We showed in this study, the both LPS-induced surface phenotype and nanostructure changes of BMDCs were suppressed by FTY720. Cellular surface topographies have been recognized as markers of both cell functional differentiation [23], [24] and maturation, specifically for DCs [22]. Heterogeneous enlargement of cell volume was frequently observed in activated-T cells [35], [36], DCs upon maturation [22], [37] and macrophages in infections [38], [39]. These changes in cell size are often accompanied by cellular height (peak to valley distance), cell surface ruffle (RMS roughness) and protrusion formation (dendrites in BMDCs) [22]–[24]. These parameters are particularly useful in quantitative evaluations on the relevance of cell structures and diseases [40]. According to the AFM observations in this study, the LPS-induced maturation of BMDCs was mitigated by FTY720 in the ways of decreasing surface particles and containing cellular enlargement. This was confirmed by the SEM assays and supported by additional information acquired from the shape index analysis. Numerous secreted vesicles, such as microvesicles (MVs), exosomes, membrane particles and apoptotic vesicles exist on different cell types; they are crucial transporters in cellular crosstalk [41], [42] (reviewed in [43]). Other than MVs (100–1,000 nm in diameter) [44], the sizes of all other vesicles are in the range of 20 to 500 nm in diameter (reviewed in [43]). As shown in the AFM and SEM results, the size of surface particles in LPS-activated BMDCs is approximately 500 to1,000 nm in diameter. Therefore, most of these structures are in MVs, according to particle sizes.

The possible suppression of MVs by FTY720, observed in this study, led us to hypothesize a drug-mediated BMDC anergy upon LPS activation. Favorably, we identified FTY720-induced overall down-regulation of cytokine production in LPS-activated BMDCs, including IL-10, IL-6, IL-12, TNF-α and MCP-1, for the first time. As a ligand of CD14, it is known that bacterial LPS induces a large amount of cytokine production, including TNF-α, IL-6, IL-8 and IL-12 [45], as well as IL-10 [46] and MCP-1 [47]. Down-regulating these cytokines by FTY720 in BMDCs is potentially significant in therapies of diseases characterized by over-activations. For example, HIV-1 infected macrophage and DCs are important suppliers of proinflammatory cytokines, including TNF-α, IL-6 and IL-1β, which are etiologically relevant to HIV-1 associated neurodegenerative diseases (HAND) [48]–[53]. We previously reported that Copexone, another approved drug on MS treatment, suppresses the neurotoxic effects of HIV-1 infected macrophages via increasing IL-10 expression and macrophage phenotype switch [52]. Interestingly, this is in agreement with the significant IL-10 up-regulation as well as Il-6 and MCP-1 down-regulation in the FTY720 alone-treated group, observed in this study.

Regarding the phenotypic switch from maturation to anergy-polarization of FTY720-treated BMDCs upon LPS activation, we posit the mechanism should partly rely on the NO intrinsic pathway. This is because as a consequence of inflammation, proinflammatory cytokines are enhancers of NO production [29], [54]. In turn, NO is a potent inducer of production of these cytokines, including TNF-α and IFN-γ [55], [56]. Under normal conditions, this positive feedback loop ends in where NO concentration becomes high enough to induce cytotoxic effects on immune cells. Therefore, reciprocal activation between NO and proinflammatory cytokines, has been identified as a mechanism to maintain inflammatory microenvironment [56]. In this study, we demonstrated an FTY720-suppressed NO production with or without LPS-treatment, which supported the above-mentioned reciprocal functionality argument. This was further supported by the FTY720-induced suppression of IL-6, TNF-α and MCP-1observed in this study.

NO is also known to induce endocytosis via cGMP pathways [57], which is partially relevant to a chemokine receptor, CCR7 [28]. This is because CCR7 is not only a migration-associated molecule, it can also stimulate protrusion formation via binding CCL19 that plays important roles in endocytosis [58]. Reduction of protrusions on DCs is a phenotypic indicator of impaired antigen presentation [59]. Favorable to these theories, we demonstrated FTY720 can down-regulate CCR7 transcription, along with impairing the phagocytotic and endocytotic capacities of BMDCs and reduction of dendrites. These mechanisms of anergy-polarized DCs were further supported by showing that FTY720 suppresses specific antigen presentation function of BMDCs, shown in this study.

The translational potential of FTY720-induced anergy-polarization, proposed in this study, is supported by recent *in vivo* investigations. As an example, local application of FTY720 to the lung was reported to contain experimental asthma via targeting at dendritic cell function [33]. This anti-allergic airway inflammation activity of FTY720 could be explained partially by a later finding of the anti-DC migration and chemokine receptor expression mitigation effects of the drug [60]. Recent studies indicated these suppressive effects of FTY720 may not lead to a systematic immunodeficiency. In a subtle study, the cellular and humoral immune responses to influenza vaccine were found unmitigated in MS patients treated with FTY720, and these response capacities are comparable to healthy subjects [61].

In conclusion, we demonstrated FTY720 can induce an anergy-polarized phenotype switch of BMDCs characterized by the impaired functions of NO production, CCR7 transcription, phagocytosis, endocytosis and antigen presentation. Upon LPS activation, this polarization became apparent by additionally exhibiting declined shape index, decreased topographical parameters as well as proinflammatory cytokines. One of the possible mechanisms of this anergy induction is FTY720 suppresses the reciprocal activation between NO and cytokines in BMDCs that mitigates CCR7 transcription, phagocytosis and endocytosis, resulting in an endpoint phenotype changes in cellular morphology and topography.

## Materials and Methods

### BMDC Preparation

All animal care and experimental procedures were performed in accordance with protocols approved by the Institutional Animal Care & Use Committee (IACUC) of Jinan University. Specific pathogen free (SPF) female Balb/c mice, 6–8 weeks old, were purchased from the Experimental Animal Center of Southern Medical University (Guangzhou, Guangdong, China). Bone marrow cells were harvested from mouse femurs and tibias, and treated with red blood cell lysis buffer (Invitrogen, Beijing, China), as described previously [39]. Cells were plated in six-well plates at 1×10^6^ cells/well and were allowed to attach to the culture surface for 6 h in RPMI complete medium, RPMI 1640 medium supplemented with 10% (V/V) fetal bovine serum (FBS), 4 mM L-glutamine, 25 mM HEPES (pH 7.2), 100 U/mL penicillin and 100 mg/mL streptomycin (all from Invitrogen), as well as 50 mM β-mercaptoethanol (Sigma-Aldrich, St Louis, MO, USA). Non-adherent cells were then removed and the residual bone marrow cells were cultured in BMDC differentiation medium, RPMI complete medium supplemented with 20 ng/mL recombinant murine GM-CSF and 20 ng/mL recombinant murine IL-4 (both from PeproTech, Rocky Hill, NJ, USA). On Day 6 post-differentiation, BMDCs were acquired and used for subsequent experiments on FTY720 (Nanjing Ange Pharmaceutical Co., Ltd, Nanjing, Jiangsu, China) and/or LPS (Sigma).

### BMDC Surface Marker Detection

BMDCs were collected and washed once with PBS, followed by antibody labeling at 4°C for 30 min, 5×10^5^ cells/sample. Cells were then washed and re-suspended in 300 µL of washing buffer (0.5% FBS and 0.05% sodium azide in PBS), and analyzed by a FACSCalibur flow-cytometer (Becton Dickinson, Beijing, China). Antibodies used were uorescein isothiocyanate (FITC)-conjugate anti-mouse CD11c mAbs, as well as phycoerythrin (PE)-conjugated anti-mouse I-A^d^, anti-mouse CD40, anti-mouse CD80 and anti-mouse CD86 mAbs (BD Biosciences Pharmingen, San Diego, CA, USA). Fluorescence activated cell sorter (FACS) data were analyzed by FlowJo software version 7.6.5 (Treestar, Inc., San Carlos, CA, USA).

### Atomic Force Microscopy

AFM observation on BMDCs was performed as previous described with minor modifications [22], [24]. In brief, BMDCs were cultured on cover glasses (Fisher Scientific, Fair Lawn, NJ, USA) pre-positioned in six-well plates. Sample preparation followed the procedure of 3 gentle washes with PBS, addition of fixative buffer (4% glutaraldehyde in PBS, 10 min), 6 gentle washes with distilled water and natural dry-off. An atomic force microscope (AUTOPROBE CP, Thermomicroscopes, Sunnyvale, CA, USA) was then employed for cell topographical observations at room temperature under tapping scanning mode. The curvature radius of the silicon nitride tip (Park Scientific Instruments, Thermomicroscopes) was close to 10 nm; and the force constant was 2.8 N/m, approximately. All AFM images were analyzed by the Proscan Image Processing software version 2.1 (Thermomicroscopes).

### Scanning Electron Microscopy

BMDCs were allowed to grow on poly-l-lysine–coated cover glasses (Fisher Scientific) before being fixed with 2.5% (v/v) paraformaldehyde in PBS for 10 min at room temperature. After 3 times PBS washes, cells were subjected to sequential dehydration with gradient ethanol (50%, 70%, 90% and 3 times 100%, respectively; 5 min for each procedure), followed by isoamyl acetate treatments (3 times, 5 min for each). Critical point drying with carbon dioxide was then performed, and samples were installed to stub holders followed by gold painting before observation with a scanning electron microscope (ESEM-30, Philips, Mahwah, NJ, USA).

### MTT Assay

3-(4,5-dimethylthiazol-2-yl)-2,5-diphenyltetrazolium bromide (MTT) assays for cytotoxicity evaluation was performed as previously described [36]. In brief, cells were incubated with MTT (5 mg/mL) solution for 4 h, followed by addition of DMSO at 100 µL per well. MTT formazan crystals were dissolved by shaking on an orbital shaker for 15 min. The absorbance was measured at 570 nm with a microplate reader (Bio-Rad, Shanghai, China).

### Apoptosis Assay

Cells were washed for two times with PBS and resuspened in 100 µL binding buffer (10 mM Hepes [NaOH pH 7.4], 140 mM NaCl, 2.5 mM CaCl2). Cells were incubated with 5 µL Annexin-V FITC (BD Pharmingen) per 5×10^5^ cells for 15 min, followed by addition of 3 µL propidium iodide (PI) buffer (50 µg/mL PI in PBS). FACS assay was then performed to detect Annxin-V at FL1 and PI at FL2 channels. Data were analyzed by FlowJo software; apoptotic cells were distinguished as Annexin-V^+^PI^−^, while necrotic cells as Annexin-V^+^PI^+^.

### Cytometric Bead Array

Cytokine secretions of BMDCs were analyzed by mouse inflammation CBA Kit (BD Pharmingen) following the procedure as previously described [36]. This technology allows detecting six cytokines (IL-6, IL-10, MCP-1, IFN-γ, TNF-α, and IL-12p70), simultaneously. Supernatants from different cell cultures were collected and aliquoted for cytokine assay. Captured bead reagent was applied to the mixture of 50 µL sample and PE-detection antibody. The reaction lasted 3 h in the dark at room temperature. All unbound antibodies were removed by addition of 1 mL wash buffer following by centrifuging. Captured beads were then re-suspended with 300 µL wash buffer and analyzed by FACS (BD). Six standard curves were acquired for each experiment by four-parameter linear fitting on the concentrations of serially diluted standards ranging from 10–5000 pg/mL.

### Real-time PCR for CCR7 mRNA Assay

The expression of CCR7 mRNA of BMDCs was detected by SYBR Green-based real-time (RT)-PCR. Total RNA was extracted from cells by trizol lysis, followed by purification with Qiagen RNeasy spin columns (Qiagen, Shanghai, China). Synthesis of cDNA was performed by using the PrimScript® Reverse Transcriptase reagent kit (TaKaRa, Dalian, Liaoning, China). A mixture of 4 µL diluted cDNA, 16 µL of SYBR Green RealMasterMix (Tiangen Biotech, Beijing) and 150 nM of each primer, was then prepared and subjected to RT PCR analysis with an ABI 7500 PCR device (Applied Biosystems, Foster City, CA, USA). Primer sequences for CCR7 included sense strand 5′-GCGGAACAAGGCCATCAAGG-3′ and antisense strand 5′-GACGGAGGCCAGGCTGTAGG-3′; Primers for β-actin included sense strand 5′-ACCGCTCGTTGCCAATAGTG-3′and antisense strand 5′-CTTCTCCATGTCGTCCCAGT-3′. The PCR procedure was 10 min/95°C, followed by 30 cycles of 94°C/15 s, 60°C/15 s and 68°C/30 s, and ended by 72°C/30 s. The relative expression level of the target genes was computed with comparative cycle threshold (Ct) method using the SDS software version 1.3.1 (Applied Biosystems).

### Intracellular NO Assay

NO formation was determined by using a Griess reagent kit (Promega, Shanghai, China) according to the manufacturer’s instructions. Briefly, 50 µL of culture supernatant was mixed with 50 µL Griess reagent and incubated at room temperature for 10 min. Optical density value was than measured at 540 nm with a microplate reader (Bio-rad). Serially diluted Griess reagent standards were used in each experiment.

### Phagocytosis Assay

BMDCs were treated with 1.0 µm carboxylate-modified yellow-green fluorescent FluoSpheres® beads (Molecular Probes, Invitrogen) for 60 min at 37°C. Cells were washed for three times with cold PBS and analyzed by FACS (BD). The phagocytotic ability was evaluated by analyzing fluorescence at FL1 channel.

### Endocytosis Assay

BMDCs were exposed to 0.1 mg/mL FITC-conjugated dextran (Sigma) for 2 h at 37°C. Cells were washed for three times with cold PBS and analyzed by FACS (BD). The endocytotic ability was evaluated by analyzing fluorescence at FL1 channel.

### Specific Antigen Presentation Ability

SPF level transgenic mice, DO11.10 strain, were purchased from Model Animal Research Center of Nanjing University (Nanjing, Jiangsu, China). DO11.10 lymphocytes were isolated and subjected to CFDA-SE (Invitrogen) staining following the procedure as described previously [36]. Cells were plated in 96-well plates at 5×10^5^ cells/well in OVA- (100 µg/mL; Sigma) or OVA peptide 323–339- (100 µg/mL; GL Biochem Ltd., Shanghai, China) containing medium. BMDCs (if added) were applied to the DO11.10 lymphocyte cultures at 5×10^4^ cells/well to present OVA or OVA peptide 323–339 to OVA-specific T cells. Activated T cells were continuously cultured for 4 d before analyzing their CFSE fluorescence intensity with FACS (BD). The lower CFSE mean fluorescence intensity (MFI) at FL-1 channel detected, the higher T cell proliferation rate it represents. A Con A-stimulated T cell proliferation model, as described previously [36], was employed as a positive control of this experiment.

### ELISA

IL-2 and IFN-γ concentrations in culture supernatants were analyzed by ELISA kits (BD Pharmingen) according to the manufacturer’s instructions. Briefly, 100 µL of serially diluted standards or samples were applied to 96-well capture plates and incubated for 2 h at room temperature. After 7 washes, detection antibody and horseradish peroxidase reagents were added into each well; plates were incubated for 1 h at room temperature before another 7 washes. 100 µL substrate was then applied to each well and the reaction was terminated by 50 µL Stop Solution provided by the kits. Absorbance was detected by a microplate reader (Bio-rad) at 450 nm.

### Statistics

Data were analyzed for statistical significance either by two-tailed Student’s *t*-test or one-way *ANOVA* with Bonferroni *post hoc* multiple comparisons using GraphPad Prism version 5.02 (GraphPad Software, Inc., San Diego, CA, USA). Significant difference was accepted when *P*<0.05.

## References

[1] Ludewig B, Freigang S, Jaggi M, Kurrer MO, Pei YC (2000). Linking immune-mediated arterial inflammation and cholesterol-induced atherosclerosis in a transgenic mouse model.. Proc Natl Acad Sci U S A.

[2] Bobryshev YV (2005). Dendritic cells in atherosclerosis: current status of the problem and clinical relevance.. Eur Heart J.

[3] Sun J, Hartvigsen K, Chou MY, Zhang Y, Sukhova GK (2010). Deficiency of antigen-presenting cell invariant chain reduces atherosclerosis in mice.. Circulation.

[4] Koltsova EK, Ley K (2011). How dendritic cells shape atherosclerosis.. Trends Immunol.

[5] Karp CL, Wysocka M, Ma X, Marovich M, Factor RE (1998). Potent suppression of IL-12 production from monocytes and dendritic cells during endotoxin tolerance.. Eur J Immunol.

[6] Wysocka M, Robertson S, Riemann H, Caamano J, Hunter C (2001). IL-12 suppression during experimental endotoxin tolerance: dendritic cell loss and macrophage hyporesponsiveness.. J Immunol.

[7] Blanco P, Palucka AK, Pascual V, Banchereau J (2008). Dendritic cells and cytokines in human inflammatory and autoimmune diseases.. Cytokine & growth factor reviews.

[8] Eriksson U, Ricci R, Hunziker L, Kurrer MO, Oudit GY (2003). Dendritic cell-induced autoimmune heart failure requires cooperation between adaptive and innate immunity.. Nat Med.

[9] Greter M, Heppner FL, Lemos MP, Odermatt BM, Goebels N (2005). Dendritic cells permit immune invasion of the CNS in an animal model of multiple sclerosis.. Nat Med.

[10] Chen M, Wang YH, Wang Y, Huang L, Sandoval H (2006). Dendritic cell apoptosis in the maintenance of immune tolerance.. Science.

[11] Chino T, Draves KE, Clark EA (2009). Regulation of dendritic cell survival and cytokine production by osteoprotegerin.. J Leukoc Biol.

[12] Soumelis V, Reche PA, Kanzler H, Yuan W, Edward G (2002). Human epithelial cells trigger dendritic cell mediated allergic inflammation by producing TSLP.. Nat Immunol.

[13] Banchereau J, Steinman RM (1998). Dendritic cells and the control of immunity.. Nature.

[14] Fujita T, Inoue K, Yamamoto S, Ikumoto T, Sasaki S (1994). Fungal metabolites. Part 11. A potent immunosuppressive activity found in Isaria sinclairii metabolite.. J Antibiot (Tokyo).

[15] Mehling M, Brinkmann V, Antel J, Bar-Or A, Goebels N (2008). FTY720 therapy exerts differential effects on T cell subsets in multiple sclerosis.. Neurology.

[16] Cohen JA, Barkhof F, Comi G, Hartung HP, Khatri BO (2010). Oral fingolimod or intramuscular interferon for relapsing multiple sclerosis.. N Engl J Med.

[17] Aktas O, Kury P, Kieseier B, Hartung HP (2010). Fingolimod is a potential novel therapy for multiple sclerosis.. Nat Rev Neurol.

[18] Brinkmann V, Billich A, Baumruker T, Heining P, Schmouder R (2010). Fingolimod (FTY720): discovery and development of an oral drug to treat multiple sclerosis.. Nat Rev Drug Discov.

[19] Muller H, Hofer S, Kaneider N, Neuwirt H, Mosheimer B (2005). The immunomodulator FTY720 interferes with effector functions of human monocyte-derived dendritic cells.. Eur J Immunol.

[20] Lan YY, De Creus A, Colvin BL, Abe M, Brinkmann V (2005). The sphingosine-1-phosphate receptor agonist FTY720 modulates dendritic cell trafficking in vivo.. Am J Transplant.

[21] Green CE, Liu T, Montel V, Hsiao G, Lester RD (2009). Chemoattractant signaling between tumor cells and macrophages regulates cancer cell migration, metastasis and neovascularization.. PLoS One.

[22] Xing F, Wang J, Hu M, Yu Y, Chen G (2011). Comparison of immature and mature bone marrow-derived dendritic cells by atomic force microscopy.. Nanoscale research letters.

[23] Cai X, Xing X, Cai J, Chen Q, Wu S (2010). Connection between biomechanics and cytoskeleton structure of lymphocyte and Jurkat cells: An AFM study.. Micron.

[24] Wang Q, Wang M, Li S, Xing X, Liu X (2011). AFM detection of mitogen-induced morphological changes in human B lymphocyte..

[25] Giordano D, Magaletti DM, Clark EA (2006). Nitric oxide and cGMP protein kinase (cGK) regulate dendritic-cell migration toward the lymph-node-directing chemokine CCL19.. Blood.

[26] Yanagihara S, Komura E, Nagafune J, Watarai H, Yamaguchi Y (1998). EBI1/CCR7 is a new member of dendritic cell chemokine receptor that is up-regulated upon maturation.. J Immunol.

[27] Marsland BJ, Battig P, Bauer M, Ruedl C, Lassing U (2005). CCL19 and CCL21 induce a potent proinflammatory differentiation program in licensed dendritic cells.. Immunity.

[28] Sanchez-Sanchez N, Riol-Blanco L, Rodriguez-Fernandez JL (2006). The multiple personalities of the chemokine receptor CCR7 in dendritic cells.. J Immunol.

[29] Bogdan C (2001). Nitric oxide and the immune response.. Nat Immunol.

[30] Sozzani S, Luini W, Borsatti A, Polentarutti N, Zhou D (1997). Receptor expression and responsiveness of human dendritic cells to a defined set of CC and CXC chemokines.. J Immunol.

[31] Adachi K, Chiba K (2008). FTY720 story. Its discovery and the following accelerated development of sphingosine 1-phosphate receptor agonists as immunomodulators based on reverse pharmacology.. Perspect Medicin Chem.

[32] Chen JK, Lane WS, Schreiber SL (1999). The identification of myriocin-binding proteins.. Chem Biol.

[33] Idzko M, Hammad H, van Nimwegen M, Kool M, Muller T (2006). Local application of FTY720 to the lung abrogates experimental asthma by altering dendritic cell function.. J Clin Invest.

[34] Heng Y, Ma Y, Yin H, Duan L, Xiong P (2010). Adoptive transfer of FTY720-treated immature BMDCs significantly prolonged cardiac allograft survival.. Transpl Int.

[35] Lin Y, Chen Y, Zeng Y, Wang T, Zeng S (2005). Lymphocyte phenotyping and NK cell activity analysis in pregnant NOD/SCID mice.. J Reprod Immunol.

[36] Ye Y, Zhang Y, Lu X, Huang X, Zeng X (2011). The anti-inflammatory effect of the SOCC blocker SK&F 96365 on mouse lymphocytes after stimulation by Con A or PMA/ionomycin.. Immunobiology.

[37] Ferlazzo G, Semino C, Melioli G (2001). HLA class I molecule expression is up-regulated during maturation of dendritic cells, protecting them from natural killer cell-mediated lysis.. Immunology letters.

[38] Glasser SW, Senft AP, Whitsett JA, Maxfield MD, Ross GF (2008). Macrophage dysfunction and susceptibility to pulmonary Pseudomonas aeruginosa infection in surfactant protein C-deficient mice.. J Immunol.

[39] Wang T, Gong N, Liu J, Kadiu I, Kraft-Terry SD (2008). Proteomic modeling for HIV-1 infected microglia-astrocyte crosstalk.. PLoS One.

[40] Lee GY, Lim CT (2007). Biomechanics approaches to studying human diseases.. Trends in biotechnology.

[41] Alaniz RC, Deatherage BL, Lara JC, Cookson BT (2007). Membrane vesicles are immunogenic facsimiles of Salmonella typhimurium that potently activate dendritic cells, prime B and T cell responses, and stimulate protective immunity in vivo.. J Immunol.

[42] Silverman JM, Clos J, Horakova E, Wang AY, Wiesgigl M (2010). Leishmania exosomes modulate innate and adaptive immune responses through effects on monocytes and dendritic cells.. J Immunol.

[43] Thery C, Ostrowski M, Segura E (2009). Membrane vesicles as conveyors of immune responses.. Nature reviews Immunology.

[44] Heijnen HF, Schiel AE, Fijnheer R, Geuze HJ, Sixma JJ (1999). Activated platelets release two types of membrane vesicles: microvesicles by surface shedding and exosomes derived from exocytosis of multivesicular bodies and alpha-granules.. Blood.

[45] Verhasselt V, Buelens C, Willems F, De Groote D, Haeffner-Cavaillon N (1997). Bacterial lipopolysaccharide stimulates the production of cytokines and the expression of costimulatory molecules by human peripheral blood dendritic cells: evidence for a soluble CD14-dependent pathway.. J Immunol.

[46] Harizi H, Juzan M, Pitard V, Moreau JF, Gualde N (2002). Cyclooxygenase-2-issued prostaglandin e(2) enhances the production of endogenous IL-10, which down-regulates dendritic cell functions.. J Immunol.

[47] Sallusto F, Palermo B, Lenig D, Miettinen M, Matikainen S (1999). Distinct patterns and kinetics of chemokine production regulate dendritic cell function.. Eur J Immunol.

[48] Persidsky Y, Zheng J, Miller D, Gendelman HE (2000). Mononuclear phagocytes mediate blood-brain barrier compromise and neuronal injury during HIV-1-associated dementia.. J Leukoc Biol.

[49] Kipnis J, Derecki NC, Yang C, Scrable H (2008). Immunity and cognition: what do age-related dementia, HIV-dementia and ‘chemo-brain’ have in common?. Trends Immunol.

[50] Kadiu I, Wang T, Schlautman JD, Dubrovsky L, Ciborowski P (2009). HIV-1 transforms the monocyte plasma membrane proteome.. Cell Immunol.

[51] Wang T, Gong N, Liu J, Kadiu I, Kraft-Terry SD (2008). HIV-1-infected astrocytes and the microglial proteome.. J Neuroimmune Pharm.

[52] Gorantla S, Liu J, Wang T, Holguin A, Sneller HM (2008). Modulation of innate immunity by copolymer-1 leads to neuroprotection in murine HIV-1 encephalitis.. Glia.

[53] Chaudhuri A, Yang B, Gendelman HE, Persidsky Y, Kanmogne GD (2008). STAT1 signaling modulates HIV-1-induced inflammatory responses and leukocyte transmigration across the blood-brain barrier.. Blood.

[54] de Vera ME, Shapiro RA, Nussler AK, Mudgett JS, Simmons RL (1996). Transcriptional regulation of human inducible nitric oxide synthase (NOS2) gene by cytokines: initial analysis of the human NOS2 promoter.. Proc Natl Acad Sci U S A.

[55] Deakin AM, Payne AN, Whittle BJ, Moncada S (1995). The modulation of IL-6 and TNF-alpha release by nitric oxide following stimulation of J774 cells with LPS and IFN-gamma.. Cytokine.

[56] Hussain SP, He P, Subleski J, Hofseth LJ, Trivers GE (2008). Nitric oxide is a key component in inflammation-accelerated tumorigenesis.. Cancer Res.

[57] Paolucci C, Rovere P, De Nadai C, Manfredi AA, Clementi E (2000). Nitric oxide inhibits the tumor necrosis factor alpha -regulated endocytosis of human dendritic cells in a cyclic GMP-dependent way.. J Biol Chem.

[58] Yanagawa Y, Onoe K (2002). CCL19 induces rapid dendritic extension of murine dendritic cells.. Blood.

[59] Kobayashi M, Azuma E, Ido M, Hirayama M, Jiang Q (2001). A pivotal role of Rho GTPase in the regulation of morphology and function of dendritic cells.. J Immunol.

[60] Marsolais D, Yagi S, Kago T, Leaf N, Rosen H (2011). Modulation of chemokines and allergic airway inflammation by selective local sphingosine-1-phosphate receptor 1 agonism in lungs.. Mol Pharmacol.

[61] Mehling M, Hilbert P, Fritz S, Durovic B, Eichin D (2011). Antigen-specific adaptive immune responses in fingolimod-treated multiple sclerosis patients.. Ann Neurol.

